# The validity of the biological eligibility criteria to antiretroviral treatment in comparison to the systematic antiretroviral treatment in a cohort of people living with the HIV in the Southern Kivu Province, Democratic Republic of the Congo

**DOI:** 10.11604/pamj.2016.25.210.9799

**Published:** 2016-12-06

**Authors:** Ildefonse Muzaliwa, Nancy Francisca Isia, Dady Yenga, Denis Kikobya, Prince Lunjwire, Philippe Bianga Katchunga

**Affiliations:** 1 Regional School of Public Health / Faculty of Medicine of the Catholic University of Bukavu, Po Box 285, Bukavu, Democratic Republic of Congo; 2 Department of Internal Medicine, Faculty of Medicine of the Catholic University of Bukavu, PoBox 285, Bukavu, Democratic Republic of Congo; 3 Institut Supérieur des Techniques Médicales de Nyangezi, Bukavu, Democratic Republic of Congo; 4 Support Center for People Living with HIV, Pharmakina Bukavu, Democratic Republic of Congo

**Keywords:** Treatment, systematic, antiretroviral, virus, immunodeficiency, southern Kivu province

## Abstract

**Introduction:**

The late screening of the majority of patients in sub Saharan region would justify a systematic antiretroviral treatment without breaking the country programs vision. he objective of this study was to determine the validity of biological eligibility criteria to antiretroviral treatment compared with systematic antiretroviral treatment in a cohort of the people living with HIV in Bukavu city.

**Methods:**

One thousand hundred and forty-nine (1149) records of people living with HIV (PLWIV) followed in three HIV health care facilities of Bukavu city were selected systematically. The ROC curve was constructed and analyzed to assess the validity of systematic antiretroviral therapy and a treatment based on WHO biological criteria.

**Results:**

The CD4 median count was 196 /mm^3^. On admission, only 17.3% of PLWHIV had a CD4≥500/mm^3^. Compared to the criteria “systematic antiretroviral treatment”, biological eligibility criteria for antiretroviral therapy, had a sensitivity of 94.9%, a specificity of 100%, an AUC of 0.97 (0.96 to 0.98) (p <0.0001) and correlation coefficient of 0.88.

**Conclusion:**

This study shows that a systematic antiretroviral treatment of seropositive patients newly detected for the HIV in sub-Saharan Africa area must be requirement outwards WHO current recommendations. Also, in order to optimize expected outcome of a systematic treatment, a systematic screening in the high-risk groups of this area should be recommended.

## Introduction

Human immunodeficiency virus (HIV) infection is a worldwide public health problem currently with 35.3 million the number of people living with HIV (PLWHIV) including 15.7 million women and 2, 1 million children less than 15 years [1]. AIDS stage is a clinical condition associated with HIV virus infection characterized by opportunistic infections. This stage is characterized by a very high mortality. Fortunately, significant progress has been made in preventing new infections and fewer deaths linked to AIDS. This progress is mainly related to a highly effective anti-retroviral treatment, which has reduced morbidity and mortality among people living with HIV by improving their immune status thus reducing several clinical manifestations [2, 3]. A few years ago, initiation of antiretroviral treatment (ART) passed first by the evaluation of some eligibility criteria to the treatment which will help the physicians to categorize people living with HIV (PLWHIV) with a view of their best ART. These criteria have evolved with time according to the aims in view. At the beginning, the objective was to treat the Acquired immunodeficiency syndrome (AIDS) stage in order to reduce mortality and as much as possible to avoid the side effects related to the antiretroviral one [4]. Currently, the treatment goal is to minimize the viral load in order to stop the infection progression and to best restore and as soon as possible the different immune functions destroyed by HIV virus. Accordingly, World Health Organization (WHO) now recommends the treatment of a positive patient newly detected for the HIV systematically regardless CD4 count [5]. Ultimately, there are no more eligibility criteria for starting an ART.

Unfortunately, sub-Saharan Africa, the area most strongly concerned by the HIV by concentrating 67% of the PLWHIV and 75% of deaths due to the AIDS [1], is characterized by a low socio-economic level of its population and a significant number of hospitals and health centers insufficiently equipped [6]. Thus, almost all of country programs of fight against the HIV in this area are supported by international organizations. These ones usually lay down guidelines in order to optimize the treatment of vulnerable firstly [7]. This vision, if it persists, could be an obstacle for implementation of WHO current recommendations. However, from the late screening viewpoint of most patients in sub-Saharan Africa usually at the advanced stage of the disease, these ones could start ART systematically without breaking the eligibility criteria of treatment theoretically more rational based on the CD4 count [6]. Thus, this work would like to assess the validity of the biological eligibility criteria to the antiretroviral treatment compared with the systematic antiretroviral treatment as recommended by WHO.

## Methods

### Study design

This retrospective study has systematically included the medical records of all adults HIV seropositive patients >15 years followed in 3 antiretroviral health care facilities of Bukavu city (Pharmakina, Bagira and General Provincial Hospital of Bukavu reference). In addition, 211 seronegative (for HIV) medical files among blood donors of the General Reference Provincial Hospital of Bukavu were also selected as a control group. The period of data collection was spread from the 1st September 2014 to 20th December 2014. All confidentiality rules have been met.

### Data collection

For each patient, socio-demographic parameters were recorded (age, sex, marital status). Then, the medical history of HIV infection was studied (the presence of opportunistic infections). Finally, the CD4 count at admission was searched.

### Study outcomes

The systematic antiretroviral treatment was to initiate ART for all adults with HIV regardless of WHO clinical stage and at any CD4 cell count [5]. Previously recommended ART for this population was to Initiate ART for all adults with HIV and a CD4 count at or below 500 cells/mm^3^, regardless of WHO clinical stage, giving priority to those with severe or advanced HIV disease (WHO clinical stages 3 or 4) or a CD4 cell count at or below 350 cells/mm^3^ [7]. In this study, On the basis of biological criteria, the PLWHIV with CD4 < 500/mm^3^ and/or if he is married (bride or divorced or widowed) and/or having an active tuberculosis were eligible to the treatment [7].

### Statistical analyses

Data are described, as frequencies or median (interquartile range), when appropriate. The distribution of the variables was tested for normality using the Kolmogorov-Smirnov test. We used Kruskal-Wallis test and the chi-square test for comparison of medians and proportions respectively. The predictive value of the current biological criteria comparatively with systematic implementation antiretroviral therapy HIV positive patients a once tested for HIV (gold standard) was studied, with ROC curves. The value of p≤0.05 defined the statistical significance. Data from participants were processed using the Epi INFO^®^ 2000 version 3.5.3 and the 12.4.0 MedCalc^®^ softwares.

## Results

### General characteristics of the studied population

Table 1 lists the general characteristics of the study population. Among 1149 PLWHIV, 428 (37.2%) were men and 721 (62.8%) were women. The median age was 42.0 (33.0 to 50.0) years. 42.1% were under 40 years, 49.2% between 40 and 59 years and only 8.7% were 60 or older. Also in the entire population of PLWHIV, 82.8% were married or had a history of marriage (divorced, widowed). 211 blood donors HIV negative constituting the control group had an average age of 37.3 ± 11.3 years. Of these, 142 were women and 68 men.

**Table 1:** General characteristics of people living with HIV studied

	Whole Group n=1149	Men n=428	Women n=721	p
**Median (IQR)**				
Age (year)	42.0 (33.0 to 50.0)	44.0 (36.0 to 53.0)	40.0 (33.0 to 49.0)	<0.0001
Duration of the disease (year)	8.0 (5.0 to 10.0)	8.0 (5.0 to 9.0)	8.0 (5.0 to 10.0)	0.0005
CD4 (/mm^3^)	225.0 (116.0 to 413.0)	180.0 (86.0 to 346.0)	242.0 (136.0 to 448.0)	<0.0001
**Frequency (%)**				
Age (year)				
<40	42.1	46.6	34.6	0.0001
40-59	49.2	46.2	54.2	0.01
≥60	8.7	7.2	11.2	0.03
CD4 (/mm^3^)				
<200	44.4	53.3	39.1	<0.0001
200-349	23.5	22.0	24.4	0.39
350-499	14.8	14.3	15.1	0.77
≥500	17.3	10.5	21.4	<0.0001
**Matrimonial status**				
Single people	17.2	16.9	17.5	0.85
Married	82.8	83.1	82.5	0.85

### Clinical settings

Table 1 reports the clinical characteristics of PLWHIV in this study. The CD4 median count was 225.0 (116.0 to 413.0)/mm^3^. 44.4% of PLWHIV had CD4 <200 /mm^3^ versus 17.3% who had CD4≥500 /mm3. Theoretically, 95.5% should be put on treatment based on biological criteria and 100% consistently.

### Validity of the eligibility criteria to treatment

Table 2 shows the results of the construction of the ROC curve. Compared with the “systematic antiretroviral treatment”, the biological criteria of treatment eligibility had a sensitivity of 94.9%, a specificity of 100% and an AUC of 0.97 (0.96 to 0.98) (p <0.0001). The correlation coefficient was 0.88.

**Table 2:** Validity of biological eligibility criteria for treatment

	AUC (IC à 95%)	Sensitivity	Specificity	Youden Index
Biological eligibility criteria	0,97 (0.96 à 0.98) p<0,0001	94.9	100.0	0.94

## Discussion

The present study shows that 95.5% of the PLWHIV were at once eligible to the ART according to the biological criteria. In the same way, compared with the systematic treatment, the biological criteria had sensitivity and specificity respectively of 95.0% and 100% as well as a coefficient of correlation of 88%. Our results confirm that a systematic antiretroviral treatment of any seropositive patient newly detected for the HIV in our area would be judicious without neither necessarily lead to a significant immediate increase in the numbers of people who actually access treatment nor breaking the country programs vision. This would be the consequence of a late HIV screening at the advanced stage (AIDS) of most of the patients who would be consequently eligible to the antiretroviral treatment if the biological criteria were applied. Our results corroborate other authors. Indeed, a meta-analysis of studies Saharan Africa showed that average CD4 presentation (N = 295,455) and initiation of treatment (N = 549,702) were low of 251 cells/µl and 152 cells/µl respectively. These averages have not significantly increased between 2002 and 2013 despite the intensification of programs against HIV [8]. From these observations viewpoint, the advantage of a systematic antiretroviral treatment in our area is certainly the reduction or the prevention of the opportunistic infections and, a small mortality reduction. However, the relevance of such attitude on the reduction of HIV transmission [9] in this area would not be optimal because of a late HIV screening, the PLWHIV remaining contagious during the long asymptomatic period characteristic of the disease. It would be ideal if proceeding to a systematic HIV screening particularly among the groups at the risk in this area in order to start systematically ART for the positive patient newly detected for the HIV. Such decision can only be issued by the country programs like ours in DR Congo. Moreover, the ART stock out conditions should be avoided.

Moreover, It is not excluded the drugs side effects or drug resistance in a medium-term related to the poor adherence in patients on ART very often due to the ART stock out. But the benefit/risk link would plead for a systematic HAART initiation in sub-Saharan Africa area. Several studies already show the benefit of an early initiation of HAART for CD4 > 500/mm3 in the new HIV infection, morbidity and mortality reduction among PLWHIV [10–13]. The major limitation of this study is to be retrospective and combining all deficiencies connected with this methodology. Furthermore, the study was not multicentric so that its results are valid for other regions of sub-Saharan Africa.

## Conclusion

This study shows that a systematic antiretroviral treatment for seropositive patients newly detected for VIH in sub-Saharan Africa area is obviousness outwards the very one WHO current recommendations. Also in order to optimize the expected outcome for a systematic ART treatment, a systematic HIV screening among the risk groups in this area should be achieved.

### What is known about this topic

- Country programs of fight against the HIV in subSaharan Africa usually lay down guidelines in order to optimize the treatment of vulnerable firstly;
- World Health Organization (WHO) now recommends the treatment of a positive patient newly detected for the HIV systematically regardless CD4 count.

### What this study adds

- A systematic antiretroviral treatment of seropositive patients newly detected for the HIV in sub-Saharan Africa area must be a requirement outwards WHO current recommendations.
